# Priorities and strategies for improving Roma women’s access to primary health care services in cases on intimate partner violence: a concept mapping study

**DOI:** 10.1186/s12939-017-0594-y

**Published:** 2017-06-07

**Authors:** Carmen Vives-Cases, Isabel Goicolea, Alison Hernández, Belen Sanz-Barbero, MCarmen Davó-Blanes, Daniel La Parra-Casado

**Affiliations:** 1 Department of Community Nursing, Public Health and Preventive Medicine and History of Science, Alicante University, Alicante, 03080 Spain; 2 CIBER de Epidemiología y Salud Pública (CIBERESP), Madrid, Spain; 3 Public Health Research Group, Alicante University, Alicante, Spain; 4 Epidemiology and Global Health Unit, Department of Public Health and Clinical Medicine, Umeå University, Umeå, Sweden; 5 National School of Public Health, Health Institute Carlos III, Madrid, Spain; 6 Department of Sociology II, Alicante University, Alicante, Spain

**Keywords:** Battered women, Healthcare, Roma population, Equity

## Abstract

**Background:**

With an explicit focus on Roma women in Spain (*Kale/Spanish Gypsies*), this study aims to integrate key informants’ opinions about the main actions needed to improve primary health care services’ and professionals’ responses to Roma women in an Intimate Partner Violence (IPV) situation.

**Methods:**

Concept mapping study. A total of 50 (brainstorming phase), 36 (sorting and rating phase) and 16 (interpretation phase) participants from Roma civil society groups, primary health care professionals and other related stakeholders (social services, academic experts and other IPV NGOs representatives) from different cities in Spain were involved in the different study phases.

**Results:**

Among the 55 action proposals generated, ten priority actions were identified through consensus as most important for improving primary health care’s response to Romani women in an IPV situation, and these included primary, secondary and tertiary prevention activities.

**Conclusion:**

Results indicated that efforts to address this challenge should take an integrated approach that reinforces the primary health care response to IPV in general, while also promoting more specific actions to address barriers to access that affect all Roma women and those who experience IPV in particular.

## Background

Intimate Partner Violence against women and girls (IPV) is an extreme manifestation of gender inequality in society and a serious violation of fundamental human rights. In European Union (EU) countries, between 20 and 25% of adult women reported having experienced IPV at some time in their lives [1]. A recent study of 54 countries showed that women have a five times greater risk of being killed by their (male) partners than men [2]. The prevention of violence against women in different forms, including IPV, is nowadays recognized as a public health priority [3].

Although IPV occurs across all social groups, ethnic minority women are in a particularly vulnerable situation due to their frequent exposure to intersecting axes of discrimination. This study proposes to focus on the particular situation of women belonging to the Roma community, the largest ethnic minority group in Europe, with an estimated population of about 10 million in the EU and several million more in countries outside the EU [4]. In Spain, the Roma population (*Kale/Spanish Gypsies*) is also the largest minority ethnic group with an estimated population of at least 700.000 people that constitutes nearly 2% of the total Spanish population [5]. Across Europe, the Roma population suffers the highest prevalence rate of discrimination, and this is also the case in Spain [6, 7]. The strength of this specific form of racism, anti-Gypsysm, points to the need for explicit focus on the Roma minority. In the case of Roma women experiencing IPV, the intersecting burden of discrimination for being Roma, female and in many cases low income makes them extremely vulnerable.

Studies about IPV in Roma women and girls are scarce. The few which have been conducted coincide in indicating alarming figures within this population. A study conducted in Bosnia and Herzegovina in 2011 estimated that 43% of Roma women had experienced past and/or current physical violence and 36% had experienced psychological abuse by their husbands [8]. In another study conducted in Turkey, it was estimated that Roma women have a three times greater risk of IPV than women in the general population [9].

Frequent exposure to poor socio-economic conditions also puts Roma women at greater risk of health problems, and consequently increases their need for accessing the health system, especially primary health care centers [10]. For Roma women who experience IPV and do not trust in authorities’ assistance, health workers may be the only point of contact with public services that can offer support and information [11]. In Spain, Roma women do access primary health care services as frequently as women from the general population and with more frequency than Roma men, in terms of consultations with general practitioners. However, they have poorer access than the general population when it comes to the sexual and reproductive health care provided by midwives and gynecologists, and preventive screenings, like mammography and Pap smear [12]. Inequities in healthcare access, including primary health care, have been described elsewhere in Europe [13–15].

Health professionals and health services represent an important resource for all women experiencing IPV [16], including those who belong to minority ethnic groups [17]. Well-trained health providers can improve IPV detection and referral to agencies specialized in violence, where intensive advocacy interventions can be provided [18, 19]. However, the literature shows that encounters between women exposed to IPV and health-care providers are not always satisfactory [20, 21]. A number of barriers that prevent individual health-care providers from responding to IPV have been pointed out: organizational barriers, time constraints, an attitude of blaming vis-à-vis women exposed to IPV, lack of training, and lack of community resources to team up with, to cite just a few [22–25].

The primary health care (PHC) approach has proved to be very effective when it comes to implementing promotive and preventive interventions against complex problems that transcend the traditional responsibilities of the health system- such as IPV [26]. This approach is characterized by person/family-centered, longitudinal, comprehensive, coordinated and community oriented care, and is facilitated by multidisciplinary teams working in primary health care centers, who act as a key interface linking ambulatory care with hospital and specialty services, and individual care with other community social services. These features of PHC may positively influence health sector responses to IPV [27].

The role of the health sector in responding to IPV is important in the context of coordinating innovative action across diverse sectors, such as social services, law enforcement, local councils and non-governmentalorganizations (NGOs), to address these endemic social inequalities [28]. The importance of this multi-sectorial focus was the starting point of this study, and we sought to incorporate the views of health professionals and representatives of different sectors in generating proposals for improving primary health care for Romani women in situations of IPV. The study was carried out in Spain, where the Roma population legally has equal access to the health system. However, in practice they may confront barriers to access related to institutional discrimination, lack of trust and information and difficulty discussing their intimate problems with strangers (especially if professionals are men, upper socio-economic class and/or belong to dominant social/ethnic group), as has been reported in different European countries [29–31].

The aim of this study was therefore to integrate key informants’ opinions about the main actions needed to improve primary health care services’ and professionals’ responses to Roma women in an IPV situation.

## Methods

A concept mapping study was carried out with women who worked in Roma civil society groups, primary health care professionals and other related stakeholders (social services, academic experts and other IPV NGO representatives) from different cities in Spain (Valencia, Alicante, Madrid, Murcia, Leon, Vigo, Huesca, Sevilla, Oviedo and A Coruña). This methodology was selected based on its capacity to enable groups of actors to visualize their ideas around an issue of mutual interest and develop common frameworks through a structured and participatory process [32]. The activities of the concept mapping process (brainstorming, rating and sorting, analysis, and interpretation of results with participants) were carried out from November 2014 to November 2015.

Potential participants from Roma civil society groups were identified through an initial internet search for Roma organizations that had promoted IPV interventions or programs which involved health professionals. A total of 28 organizations were identified and of these twelve (43%) participated in brainstorming, and eight continued participating in the rating and sorting and interpretation phases. The participants representing each organization were individuals who the organizations indicated had the most work experience with the topic.

Recruitment of primary health care professionals began with contacting the managers responsible for coordinating IPV activities in the health sector in the 17 autonomous regions of Spain, who had participated in a previous study carried out by some of the authors [33]. Of the 26 managers contacted, twelve agreed to participate (ten medical doctors and two social workers), while the others indicated that they were not available or were currently working in other topics. In addition, we contacted professionals working in primary health care centers (PHCCs) with experience with IPV cases who had been identified in another previous study [27] or identified by other participants using snowball sampling technique. Through these approaches, a total of twenty contacts were made, of whom 14 agreed to participate (four medical doctors, three midwives, three psychologists, two social workers and two pediatricians). Of the total 26 health professional participants, 26 participated in brainstorming, 15 in sorting and rating (five from management, ten from PHCCs), and six in the interpretation phase (one from management, five from PHCCs).

The remaining twelve participants from women’s associations, violence associations, health agents and social workers from different public services related to IPV were contacted by telephone and/or e-mail based on suggestions from the other participants. In this case, all those contacted agreed to participate, and twelve participated in brainstorming, eleven in sorting and rating, and two in the interpretation phase (Table 1).

**Table 1:** Participants representing different groups in each phase of the concept mapping process

	Roma associations	Health professionals	Other IPV-focused organizations	Total participants
Phase 1: Brainstorming	12	26	12	50
Phase 2: Rating and Sorting	8	15	11	36
Phase 3: Interpretation	8	6	2	16

Spain, 2016

In the brainstorming phase, participants provided ideas on how to improve the health sector response to IPV in the Romani population, drawing on their experience and expertise. Participants were recruited via email invitation followed by telephone interviews which were recorded and later transcribed. Participants were asked to answer the following focus question: *In what aspects should primary health care services and professionals improve in order to better respond to Roma women in situations of IPV?* This first step was carried out from November 2014 to March 2015. Participant interviews yielded a total of 81 idea statements. The transcriptions of the idea statements provided by participants were reviewed by the research team and after eliminating repetitions, there were a total of 55 unique action proposals, which were assessed in the following phase.

In the rating and sorting phase, participants assessed the action proposals of the group as a whole by rating their importance and feasibility and sorting them into groups based on the similarity of the ideas. Three on-line questionnaires (one for sorting and two for rating) were designed with the support of the Concept Systems software [34] using the statements collected in the previous step. Participants in the brainstorming phase were invited via email and telephone to complete the questionnaires and 72% responded. For the sorting questionnaire, the participants organized the statements into groups that had meaning for them. In the rating questionnaires, the participants rated the importance and feasibility of each of the statements on a scale from 1 to 6, from lowest to highest importance/feasibility. The phase of rating and sorting was performed from April to June 2015.

Sorting data was analyzed using multi-dimensional scaling to generate a point map, where statements are plotted based on the number of times participants grouped them together, with those that were frequently grouped together positioned close to each other. Hierarchical cluster analysis was used to generate cluster maps where statements are aggregated into clusters based on their proximity to each other in the point map. Maps depicting how statements were grouped into cluster solutions ranging from two to ten clusters were evaluated, and the most appropriate number of clusters was determined through discussion among the research team. Successive levels of clustering were evaluated based on their conceptual coherence and the value of precision offered at each level. Averages of the importance and feasibility ratings for each statement were calculated and the combined importance and feasibility scores were used to identify priority idea statements. The cluster map and prioritized list of statements served as the results to be reviewed by participants in the interpretation phase. An additional step of identifying broader domains connecting sub-groups of clusters focused on similar areas of action was conducted by the research team after the cluster map was reviewed by participants.

The final phase of interpretation of results was carried out in a participatory workshop held at the University of Alicante in October 2015, and 44% of the participants in the previous phase of sorting and rating were able to attend. The objective of the workshop was to reach consensus among participants on ten priority action proposals to improve the primary health care response to Roma women in an IPV situation based on review of the results of the previous phases. The workshop was facilitated by part of the research team (C Vives-Cases, D La Parra and MC Davo), and it began with a review of the cluster map and open discussion of proposals of names for the clusters. Then participants worked in three small groups, and they were asked to review and discuss the importance and feasibility scoring of the 55 action proposals and identify the most essential proposed actions based on scores as well as their own criteria. The groups then presented their lists of most essential actions in a plenary discussion. The groups’ suggestions were compiled and the participants were then asked to rate each of the most essential proposed actions (on a scale from 1 to 6). These results were processed and another plenary discussion followed where they developed consensus on the final set of ten priority action proposals, which was composed of the statements that had the highest ratings and was confirmed by the workshop participants.

## Results

**Fig. 1:**
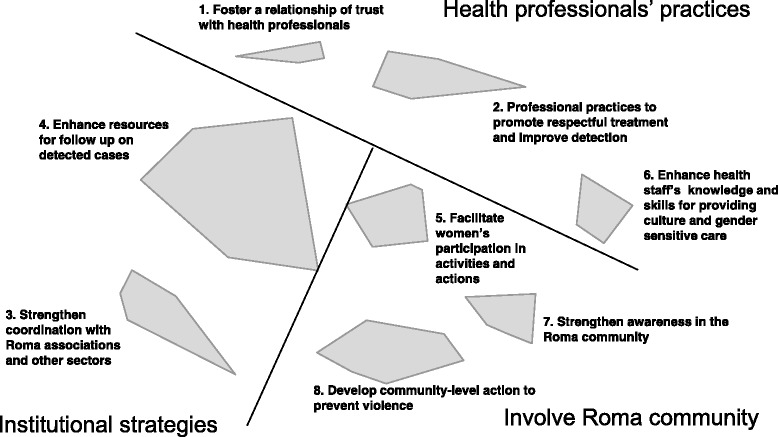
Thematic clusters of participants’ proposals to improve primary care responses to violence against Roma women

Participants proposed a total of 55 actions to improve primary health care services’ and professionals’ responses to Romani women seeking help due to their situation of IPV (Table 2). The final clustering solution generated eight clusters or thematic areas of action: 1) Foster a relationship of trust with health professional; 2) Professional practices to promote respectful treatment and improve detection; 3) Strengthen coordination with Roma associations and other sectors; 4) Enhance resources for follow up on detected cases; 5) Facilitate women’s participation in activities and actions; 6) Enhance health staff’s knowledge and skills for providing culture and gender sensitive care; 7) Strengthen awareness in the Roma community; and 8) Develop community-level action to prevent violence. Figure 1 depicts the final cluster solution. The varying sizes of the clusters reflect the tightness of the conceptual coherence of the actions they contain, while their proximity to each other reflects perceived relationship between the actions they contain. Based on review of the proximity and the content of the clusters, we further identified sub-groups of clusters that shared focus on similar domains of action. These domains of action included improving health professionals’ practices (Clusters 1, 2, 6), and strengthening the primary care system’s response at the institutional level (Clusters 3, 4) and at the community level (Clusters 5, 7, 8).

**Table 2:** Recommended Actions to improve primary health care responses to violence against Romani women

Cluster	Statement	Importance	Feasibility
Actions to enhance health professionals’ capacity to respond to battered Roma women			
1. Foster a relationship of trust with health professionals	Ensure confidentiality for women who decide to seek help	5,91	5,7
	Facilitate the accompaniment of women who are in situations of abuse if they want to file a report to help minimize risks, advise them, give them support, generate trust and facilitate referrals to other professionals	5,32	4,27
	Help women see that the health professionals are going to be there for them, and not tell them that they have to file a report or coerce them	5,2	4,55
	Talk with the women so that they know that they can get away from the violence and that their relationship with their partner isn’t healthy	4,94	3,91
2. Professional practices to promote respectful treatment and improve detection	Give unprejudiced and equal treatment to everyone	5,37	4,52
	Be attentive to non-verbal communication, fear and submissiveness	5,34	4,64
	Adapt language in the consultation so that it is easy to understand	5,31	5,06
	Be attentive to indirect indicators, such as injuries in hidden sites, and children missing a lot of school	5,31	4,76
	Understand the cycle of violence and acquired defenselessness and don’t assume that it is useless to make an effort because they will just go back	5,12	4,15
	Be attentive to the psychosocial indicators of gender violence, which are very high in Roma women	5,06	4,55
	Don’t take quick action without thinking through the repercussions that the interventions can have	4,86	4,33
	Be attentive to hyper-frequent visits by Roma women	4,69	4,3
	Understand their limits in the tolerance of violence	4,37	3,91
	Be aware of the barrier to reporting posed by the informal cultural tradition in the Roma community that custody be given to the father	4,21	4,18
6. Enhance health staff’s knowledge and skills for providing culture and gender-sensitive care	Improve and expand training in gender-based violence for all staff in the health centers, including administrative and reception staff, to know how to provide security and support in cases of abuse	5,6	4,42
	Establish mandatory training for health professionals starting from the university regarding vulnerable groups	5,49	4,15
	Provide sensitivity training about the social situation and Roma culture (dress, roles, etc.) to eliminate prejudices and stereotypes about this population	5,29	4,27
	Hold clinical rounds about prejudices and gender-based violence for health professionals – doctors and non-doctors	5,26	4,67
	Develop training about inter-personal intervention techniques (questions, attitudes) to be able to reach the women better	5,14	4,82
	Provide education about how the women experience intimate partner violence, how they suffer and how they express it	5,14	4,18
	Involve Roma associations in the training of staff in the health centers	4,37	3,45
Actions to strengthen primary health care’s responses at the institutional level			
3. Strengthen coordination with Roma associations and other sectors	Promote coordination with education to put prevention first: work with gender equality in the school, including education on sexism and gender roles	5,49	4,79
	Coordinate with people who work with Roma associations as social educators and social workers	5,24	4,79
	Foster institutional support and the involvement of managers to develop relationships with Roma associations and carry out activities together	5,23	4,15
	Make a list of the social resources available in the nearby area where they can be referred or can go for consultation	5	5,36
	Facilitate the inclusion of communication with associations in the professional duties of the doctor so that they can dedicate part of their time to this	4,69	3,21
	Coordinate with specialists (e.g. mental health, obstetrics) to detect cases during delivery care	4,6	4,24
	Coordinate with Roma health mediators from other cities so that they are not from the family of the aggressor	4,53	3,36
4. Enhance resources for follow up on detected cases	Coordinate with pediatrics when gender-based violence cases are detected to monitor the effects in the children	5,6	5,33
	Offer the women resources that are available even if they have not filed a report– shelters, legal support, economic help, etc.	5,51	4,15
	Inform the women about what resources they have and how to access them	5,49	5,12
	Reinforce the role of the social worker in the detection of cases	5,17	5
	Develop programs and protocols that take into account the unique characteristics of this collective group	5	4,7
	Reinforce the figure of the community nurse	5	4,48
	Hire professional Roma women to work with affected women	4,86	4,36
	Institutional measures, such as hiring more staff, to make it possible to extend consultations by a half hour when cases are detected	4,69	2,88
	Stabilize staffing, so that they stay in the same neighborhoods and don’t have temporary contracts, to generate trust	4,63	2,61
Actions to strengthen primary health care’s responses at the community level			
5. Facilitate women’s participation in activities and actions	Encourage Roma women to use antenatal health care and education services, and thus increase opportunities to detect violence during pregnancy	5	4,45
	Start therapy groups for women in the centers where they are referred	4,97	3,97
	Facilitate Roma women’s access to health centers, providing alternative entry points to primary health care	4,65	3,55
	Encourage the training of Roma health professionals (nurse, social worker)	4,53	2,79
	Promote home visits	4,11	3,67
7. Strengthenawareness in the Roma community	Provide training to Roma health agents and the Roma community associations on gender-based violence	5,4	4,21
	Work to develop the autonomy of Roma women	5,37	4,21
	Carry out trainings and workshops with participation from social services and Roma associations about empowerment, self-esteem, interpersonal relationships, and prevention of gender-based violence	5,34	4,61
	Outreachclinics to bring primary care providers closer to the Roma populations	4,89	4,18
	Make health center’s activities about gender-based violence more attractive for Romani women	4,8	3,64
	Promotional materials (like posters and brochures) about prevention and awareness of gender-based violence adapted to the Roma reality	4,44	4,61
8. Develop community-level action to prevent violence	Promote Roma women’s participation in the design of interventions or programs for prevention of gender-based violence	5,49	4,24
	Promote community health projects with activities and interventions about empowerment in vulnerable neighborhoods with Roma population	5,43	4,42
	Work with Roma health mediators	5,29	4,42
	Work with male Roma health mediators to transmit different values to the men	5,26	3,52
	Involve health professionals from all health centers in community health programs for prevention of gender-based violence	5,09	3,61
	Carry out activities with the Roma population to improve their trust in health staff	4,97	3,79
	Involve key actors from the Roma community (respected people, artists, athletes, professionals, social activists, the church) to make prevention campaigns	4,89	4,12

Spain, 2016

Clusters with actions addressed to health professionals (clusters 1, 2, and 6) included recommendations of practices to improve their interaction with Roma women during the consultation, to be attentive to signs of abuse and provide them with support, and training to enhance their cultural sensitivity and better understand the IPV vulnerability of Roma women. The clusters focused on institutional strategies (clusters 3 and 4) contained actions to strengthen the coordination between the primary health system and other relevant sectors, including social services and Roma associations, and also provided health professionals with facilitating institutional conditions and tools. The three clusters where actions are addressed to the community level (clusters 5, 7, 8) include recommended actions to develop strategies to facilitate Roma women’s access to IPV services, increase women’s and Roma population’s active participation in responses, and explore opportunities for primary prevention strategies working with the Roma community.

Table 2 action proposals to improve primary health care responses to violence against Roma women and average importance and feasibility rating scores. Spain, 2016.

The final set of ten priority action proposals reached through consensus among the participants in the interpretation workshop is presented in Table 3. Among the ten actions, four are from the cluster “Enhance health staff’s knowledge and skills for providing culture and gender sensitive care”, and included training in topics related to the situation of social disadvantage and discrimination against the Roma population, prejudice and gender-based violence, and sensitive treatment in cases of abuse. Other prioritized actions focus on strengthening coordination between primary health services and Roma associations and with schools to promote prevention of gender-based violence; institutional efforts to improve provision of information on resources available for protection; and community level actions to empower and promote the autonomy of Roma women. While there were some proposals that received higher average scores in the rating activity that were not included, the ten priority actions identified in the interpretation workshop represent the outcome of the participants’ collaborative evaluation of the rating results.

**Table 3:** Priority actions to reinforce primary health sector response to violence against Roma women and corresponding level of prevention

Action statements prioritized by participants^a^	Level of prevention
Establish mandatory training for health professionals starting from the university regarding vulnerable groups.	SECONDARY
Promote coordination with education to put prevention first: work with gender equality in the school, including education on sexism and gender roles.	PRIMARY
Offer the women resources that are available even if they have not filed a report– shelters, legal support, economic help, etc.	TERTIARY
Provide sensitivity training about the social situation and Roma culture (dress, roles, etc.) to eliminate prejudices and stereotypes about this population.	SECONDARY
Foster institutional support and the involvement of managers to develop relationships with Roma associations and carry out activities together.	PRIMARY
Promote community health projects with activities and interventions about empowerment in vulnerable neighborhoods with Roma population.	PRIMARY
Work to develop the autonomy of Roma women.	PRIMARY
Improve and expand training in gender-based violence for all staff in the health centers, including administrative and reception staff, to know how to provide security and support in cases of abuse.	SECONDARY
Carry out trainings and workshops with participation from social services and Roma associations about empowerment, self-esteem, interpersonal relationships, and prevention of gender-based violence.	PRIMARY
Hold clinical rounds about prejudices and gender-based violence for health professionals – doctors and non-doctors.	SECONDARY

^a^The order of the statements reflects the ratings assigned during the prioritization process, with the highest rated statement listed first

Spain, 2016

## Discussion

Our participants agreed on a list of ten priority actions to improve primary health care services’ and professionals’ responses to Roma women in an IPV situation that were related to primary, secondary and tertiary prevention activities. Half of this list was composed by IPV primary prevention activities and only one refers to the tertiary prevention level. These ten priority actions were agreed on by our key informants from a total of 55 statements related to improving health professionals’ practices and strengthening the primary care system’s response to IPV in general and to IPV among Roma women, in particular.

Many of the proposed primary health care improvement actions are expected to be developed outside health care centers and involve other sectors such as the university and the school, Roma associations and social services. Participants agreed about the importance of involving primary health care professionals in actions to improve social participation of Roma population, to increase their awareness about IPV and trust toward the health system, and to eliminate prejudices against Roma population among health professionals. The prioritized improvement proposals support a community-based primary health care approach, including work to develop Roma women’s autonomy and coordinate with schools on gender equality education. The community orientation of primary health care services is at the root of the PHC approach [26] and in Europe as well as Spain, it has been ratified by policies and guidelines [35, 36]. However, community-orientation is commonly recognized as one of the most challenging attributes to achieve when implementing a PHC approach within health systems [37–39]. Previous studies in Spain show that this attribute has been not in focus within the Spanish PHC in general, and even less in regards to IPV [33].

A second important group of improvement proposals in this study refers to primary health care professionals’ training in gender-based violence and IPV in general, and Roma population culture in particular. The relationship between training and increased readiness to manage IPV among healthcare professionals has been extensively evidenced [40]. What is particularly noteworthy is participants’ recognition of healthcare professionals’ need to know more about Roma population issues, in order to be able to tackle the Roma women’s possible lack of trust as well as other communication problems that may hinder Roma women’s acceptance of their role in dealing with IPV and other health issues [41].

Only five of the total 55 improvement proposals and one among the 10 identified priority actions referred to how to manage detected IPV cases. These actions focused on tertiary prevention referred to the need to facilitate access to support mechanisms, regardless of whether a report of the case was filed, avoiding the controversial issue of mandatory reporting. Current legislation in Spain mandates that healthcare professionals must issue a report of injuries, but it also specifies that the professional should ensure the safety of the woman and inform her, which can lead to the decision to not make the report [42]. According the WHO guidelines, mandatory reporting to the police by healthcare professionals is not recommended and incidents should be reported to the police only if the woman chooses [43].

Nearly all proposed actions to enhance health professionals’ capacity to respond to Roma women in an IPV situation are close to WHO’s recommendations about how health systems should tackle cases of violence against women in general [43]. Our results share the emphasis that the WHO recommendations place on women-centred care and professionals’ preparation for managing cases of IPV. However, in our study, cultural sensitivity also receives strong emphasis, and the importance of this issue is not mentioned in the WHO guidelines as a consideration in the provider-user interaction nor in the training of healthcare professionals. Another difference is that the WHO guidelines emphasize that clinical care for IPV should be implemented hand in hand with care for women exposed to sexual violence [43], while no specific mention to sexual violence emerged in our study. Furthermore, in line with this clinical focus, the guidelines draw very little from evidence of effectiveness in empowerment and community work, and the studies mentioned from these areas focus on shelters and support groups that are not connected to health services. This contrasts with the emphasis our participants placed on community work and the direct connection between the primary health care centers and the community. One explanation for these differences is that the WHO guidelines are not limited to primary care, but also address other health services including emergencies, hospital and specialist care.

The following limitations should be taken into account in interpreting the results of this study. Participants in the study were selected based on their knowledge of the Roma population. This represents a potential source of bias, as their ideas for action did not represent the views of the average health professional in the Spanish health system. However, this bias can be positive as it favors the inclusion of expert knowledge and the exclusion of stereotypes and prejudices, which contributes to the validity of the findings. Furthermore, as participants’ proposals were based on their own professional experience, they do not necessarily align with best practices upheld in the literature. Regarding the ten priority actions identified through this study, it should be considered that this list was reached through a process of consensus which implies that ideas of great value can be excluded because they were not understood or supported by the majority. Finally, the proposals for action generated through the study reflect the framing of the focus question at the core of the concept mapping process, which addressed the specific situation of Romani women. However, we also obtained a good representation of strategies oriented to improving the health system response to IPV for all women, particularly those who face greater barriers to access.

## Conclusion

Priority actions for improving the health system’s response to Roma women in situations of IPV identified through this study were reached through a participatory process involving Roma associations, health professionals and IPV experts. The efforts to address this challenge should take both a general health equity approach to reinforce the primary health care response to gender-based violence and a more culturally-specific approach to address Roma women’s needs. The need to strengthen the primary care system’s role in coordinating preventive strategies in collaboration with other sectors and Roma associations, and to improve the training of health professionals and staff in sensitivity towards the Roma culture were also emphasized. Actions to improve management of detected cases were also described, though to a lesser extent compared to actions related to professional training and participatory work with the Roma community. The next phase of research should focus on implications for implementation of the actions proposed.

## References

[1] Martinez M, Schröttle M. State of European research on the prevalence of interpersonal violence and its impact on health and human rights. In: Co-ordination Action on Human Rights Violations (CAHRV). European Union. 2006. http://www.cahrv.uni-osnabrueck.de/reddot/CAHRVreportPrevalence%281%29.pdf. Accessed 14 Mar 2017

[2] Alvazzi del Frate A. When the Victim is a Woman. In: Geneva Declaration Secretariat. 2011. http://www.genevadeclaration.org/fileadmin/docs/GBAV2/GBAV2011_CH4.pdf. Accessed 14 Mar 2017

[3] World Health Organization/London School of Hygiene and Tropical Medicine. Preventing intimate partner and sexual violence against women: taking action and generating evidence. In: World Health Organization. Geneva. 2010. http://www.who.int/violence_injury_prevention/publications/violence/9789241564007_eng.pdf. Accessed 14 Mar 2017.

[4] European Commission. An EU framework for national Roma integration strategies up to 2020. In: European Commission. Brussels. 2011. http://ec.europa.eu/justice/policies/discrimination/docs/com_2011_173_en.pdf. Accessed 14 Mar 2017.

[5] La Parra D, Gil-Gonzalez D, Jimenez A (2013). Social exclusion processes and the health status of the Roma people in Spain. Gac Sanit.

[6] Arza J, Carrón J (2015). Comunidad gitana: la persistencia de una discriminación histórica. OBETS Revista de Ciencias Sociales.

[7] European Union Minorities and Discrimination Survey. Main Results Report. In: European Union Agency for Fundamental Rights. Vienna. 2009. http://fra.europa.eu/sites/default/files/fra_uploads/663-FRA-2011_EU_MIDIS_EN.pdf. Accessed 14 Mar 2017.

[8] Prava za sve/Inicijativa i civilna akcija. Report within the project: Roma women for like without violence. Response of institutions to domestic violence. In: International Council of Voluntary Agences, Rights for All Bosnia & Herzegovina and United Nations Trust Fund to End Violence Against Women. Sarajevo. 2011. http://www.rightsforall.ba/publikacije-en/docs-en/Romkinje_za_zivot_bez_nasilja-ENG.pdf. Accessed 14 Mar 2017.

[9] Tokuc B, Ekuklu G, Avcioglu S (2010). Domestic violence against married women in Edirne. J Interpers Violence.

[10] Corsi M, Crepaldi C, Lodovici MS, Boccagni P, Vasilescu C (2010). Ethnic Minority and Roma Women in Europe: A Case for Gender Equality: Synthesis Report.

[11] World Health Organization. Violence Against Women: A Priority Issue. WHO Violence against women information pack 2001. In: World Health Organization. Women's Health and Development Unit. Geneva. 2001. http://www.popline.org/node/532065. Accessed 14 Mar 2017.

[12] Daniel La Parra Casado et al. Hacia la equidad en salud. Disminuir las desigualdades en una generación en la comunidad gitana. Estudio comparativo de las encuestas nacionales de salud a población gitana y población general de España, 2006. In: Consejo estatal del pueblo gitano. Ministerio de sanidad y política social. Fundación secretariado gitano. Madrid. 2009. http://www.msc.es/profesionales/saludPublica/prevPromocion/promocion/desigualdadSalud/docs/equidadSalud_05Mayo.pdf. Accessed 15 Mar 2017.

[13] Jarcuska P, Bobakova D, Uhrin J, Bobak L, Babinska I, Kolarcik P (2013). Are barriers in accessing health services in the Roma population associated with worse health status among Roma?. Int J Public Health.

[14] Földes ME, Covaci A (2012). Research on Roma health and access to healthcare: state of the art and future challenges. Int J Public Health.

[15] Cook B, Wayne GF, Valentine A, Lessios A, Yeh E (2013). Revisiting the evidence on health and health care disparities among the Roma: a systematic review 2003–2012. Int J Public Health.

[16] Sixty-Seven World Health Assembly, Resolution WHA 67.13. Strengthening the role of the health system in addressing violence, in particular against women and girls, and against children. In: World Health Organization. Geneva. 2014. http://apps.who.int/gb/ebwha/pdf_files/WHA67/A67_ACONF1Rev1-en.pdf. Accessed 15 Mar 2017.

[17] World Health Organization. How health systems can address health inequities linked to migration and ethnicity. In: WHO Regional Office for Europe. Copenhagen. 2010. http://www.euro.who.int/__data/assets/pdf_file/0005/127526/e94497.pdf. Accessed 15 Mar 2017.

[18] Feder G, Davies RA, Baird K, Dunne D, Eldridge S, Griffiths C (2011). Identification and Referral to Improve Safety (IRIS) of women experiencing domestic violence with a primary care training and support programme: a cluster randomised controlled trial. Lancet.

[19] Ramsay J, Carter Y, Davidson L, Dunne D, Eldridge S, Feder G (2009). Advocacy interventions to reduce or eliminate violence and promote the physical and psychosocial well-being of women who experience intimate partner abuse. Cochrane Database Syst Rev.

[20] Edin KE, Dahlgren L, Lalos A, Högberg U (2010). “Keeping Up a Front”: Narratives About Intimate Partner Violence, Pregnancy, and Antenatal Care. Violence Against Women.

[21] Pratt-Eriksson D, Bergbom I, Lyckhage ED (2014). Don’t ask don’t tell: Battered women living in Sweden encounter with healthcare personnel and their experience of the care given. Int J Qual Stud Health Well-being.

[22] Gutmanis I, Beynon C, Tutty L, Wathen CN, MacMillan HL (2007). Factors influencing identification of and response to intimate partner violence: a survey of physicians and nurses. BMC Public Health.

[23] Kirst M, Zhang YJ, Young A, Marshall A, O’Campo P, Ahmad F (2012). Referral to Health and Social Services for Intimate Partner Violence in Health Care Settings: A Realist Scoping Review. Trauma Violence Abuse.

[24] Ramsay J, Rutterford C, Gregory A, Dunne D, Eldridge S, Sharp D (2012). Domestic violence: knowledge, attitudes, and clinical practice of selected UK primary healthcare clinicians. Br J Gen Pract.

[25] Beynon CE, Gutmanis IA, Tutty LM, Wathen CN, MacMillan HL (2012). Why physicians and nurses ask (or don’t) about partner violence: a qualitative analysis. BMC Public Health.

[26] Starfield B, Shi L, Macinko J (2005). Contribution of primary care to health systems and health. Milbank Q.

[27] Goicolea I, Vives-Cases C, Hurtig AK, Marchal B, Briones-Vozmediano E, Otero-Garcia L (2015). Mechanisms that Trigger a Good Health-Care Response to Intimate Partner Violence in Spain. Combining Realist Evaluation and Qualitative Comparative Analysis Approaches. PLoS ONE.

[28] Garcia-Moreno C, Hegarty K, d’Oliveira AF, Koziol-McLain J, Colombini M, Feder G (2015). The health-systems response to violence against women. Lancet.

[29] European Agency for Fundamental Rights (FRA), OSCE High Commissioner on National Minorities and the Council of Europe’s Migration and Roma/Gypsy Division. Breaking the Barriers - Romani Women and Access to Public Health Care. Luxembourg. 2003. http://fra.europa.eu/sites/default/files/fra_uploads/180-ROMA-HC-EN.pdf. Accessed 15 Mar 2017.

[30] European Agency for Fundamental Rights (FRA). European Union Minorities and Discrimination Survey. Data in focus report. The Roma. In: European Union agency for Fundamental rights. Budapest. 2009. http://fra.europa.eu/sites/default/files/fra_uploads/413-EU-MIDIS_ROMA_EN.pdf. Accessed 15 Mar 2017.

[31] Hanssens LGM, Devisch I, Lobbestael J, Cottenie B, Willems S (2016). Accessible health care for Roma: a gypsy’s tale a qualitative in-depth study of access to health care for Roma in Ghent. Int J Equity Health.

[32] Kane M, Trochim WMK (2007). Concept Mapping for Planning and Evaluation.

[33] Goicolea I, Briones-Vozmediano E, Öhman A, Edin K, Minvielle F, Vives-Cases C (2013). Mapping and exploring health systems’ response to intimate partner violence in Spain. BMC Public Health.

[34] Concept Systems Incorporated: Concept Systems Software. http://www.conceptsystems.com/(2013). Accessed 24 Feb 2011.

[35] Allen J, Gay B, Crebolder H, Heyrman J, Svab I, Ram P. The European Definition of General Practice/Family medicine. In: SemFYC, EURACT. Barcelona. 2005. http://www.woncaeurope.org/sites/default/files/documents/Wonca%20definition%20spanish%20version.pdf. Accessed 15 Mar 2017.

[36] Manuel Martin G. Treinta años del Sistema Sanitario español (1981–2011). La atencion primaria, antes y despues de la Ley general de Sanidad. In: FADSP. Madrid. 2011. http://www.fadsp.org/components/com_booklibrary/ebooks/Treinta%20a%C3%B1os%20del%20sistema%20sanitario.pdf. Accessed 14 Mar 2017.

[37] Briones-Vozmediano E, Maquibar A, Vives-Cases C, Ohman A, Hurtig AK, Goicolea I (2015). Health-Sector Responses to Intimate Partner Violence: Fitting the Response Into the Biomedical Health System or Adapting the System to Meet the Response?. J Interpers Violence.

[38] Mosquera PA, Hernández J, Vega R, Martínez J, Sebastián MS (2013). Performance evaluation of the essential dimensions of the primary health care services in six localities of Bogota–Colombia: a cross-sectional study. BMC Health Serv Res.

[39] Macinko J, Almeida C, De Sá PK (2007). A rapid assessment methodology for the evaluation of primary care organization and performance in Brazil. Health Policy Plan.

[40] Zaher E, Keogh K, Ratnapalan S (2014). Effect of domestic violence training: systematic review of randomized controlled trials. Can Fam Physician.

[41] Tanahashi T (1978). Health service coverage and its evaluation. Bull World Health Organ.

[42] López Rodríguez RM, Peláez Moya S. Protocolo común para la actuación sanitaria ante la violencia de género. In: Ministerio de Sanidad, Servicios Sociales e Igualdad. Madrid. 2012. http://www.violenciagenero.msssi.gob.es/profesionalesInvestigacion/sanitario/docs/PSanitarioVG2012.pdf. Accessed 15 Mar 2017.

[43] World Health Organization. Responding to intimate partner violence and sexual violence against women. WHO clinical and policy guidelines. In: World Health Organization. Geneva. 2013. http://www.who.int/reproductivehealth/publications/violence/9789241548595/en/. Accessed 15 Mar 2017. 24354041

